# Psilocybin therapy and anorexia nervosa: a narrative review of safety considerations for researchers and clinicians

**DOI:** 10.1186/s40337-024-01005-z

**Published:** 2024-04-24

**Authors:** Amanda E. Downey, Anita V. Chaphekar, Joshua Woolley, Marissa Raymond-Flesch

**Affiliations:** 1 Department of Pediatrics, University of California, San Francisco, CA USA; 2 Department of Psychiatry and Behavioral Sciences, University of California, San Francisco, CA USA; 3 San Francisco Veteran’s Affairs Medical Center, San Francisco, CA USA; 4 Philip R. Lee Institute for Health Policy Studies, University of California, San Francisco, CA USA

**Keywords:** Psilocybin, Anorexia nervosa, Clinical trial, Adverse effects

## Abstract

**Background:**

Clinical trials using psilocybin therapy to treat anorexia nervosa (AN) are currently underway. The safety and tolerability of psilocybin is of utmost importance in individuals with AN who may present unique medical vulnerabilities. The purpose of this review is to describe how the common physiologic adverse effects of psilocybin may impact medical complications experienced by individuals with AN in clinical trials of psilocybin therapy.

**Main body:**

The physiologic underpinnings of common adverse effects following psilocybin administration are described, including tachycardia, hypertension, electrocardiogram changes, nausea, headache, and lightheadedness. These anticipated physiologic changes are described in relation to the common medical correlates seen in individuals with AN. Risk mitigation strategies for each adverse effect are proposed.

**Conclusion:**

Early evidence suggests that psilocybin therapy is well-tolerated in individuals with AN. Understanding the unique medical complications of AN, and how they may be impacted by common physiologic adverse effects of psilocybin administration, leads to tailored risk mitigation strategies to enhance safety and tolerability of this novel intervention.

## Abstract (pa)

Clinical trials using psilocybin therapy to treat anorexia nervosa are underway. Individuals with anorexia nervosa have unique medical vulnerability which may overlap with the physiologic effects caused by psilocybin administration. The safety and tolerability of psilocybin therapy in this population can be optimized through the risk mitigation strategies proposed in this review.

## Background

Individuals with anorexia nervosa (AN) experience serious and life-threatening medical complications as well as higher healthcare costs, inferior quality of life, and higher mortality compared to the general population [1]. While weight restoration is a primary treatment target to reduce the effects of starvation, weight gain alone does not always yield psychological symptom improvement [2, 3], leaving patients in a physiologically vulnerable state. The most efficacious and well-researched treatment for young people with AN, family-based treatment, yields recovery in only 38% of those treated [4, 5]. Furthermore, a large meta-analysis by Murray et al. showed that specialized AN psychotherapy modalities (cognitive behavioral therapy, parent focused therapy, acceptance and commitment therapy, among others) are not more effective than comparator treatments (placebo, supportive management) in curtailing the psychological symptoms of AN [6]. Unlike psychopharmacologic interventions in other mental health conditions, few medications have emerged as helpful in improving AN cognitions, and no medication has been proven useful in successfully treating AN [7, 8]. This sub-optimal response to evidence-based treatments yields chronic and unacceptable medical and psychological suffering. Thus, novel treatments are urgently needed to address this serious and increasingly prevalent condition.

Psilocybin is a tryptamine and indolylalkylamine and the prodrug of the active compound of hallucinogenic mushrooms [9–11]. Once ingested, psilocybin is rapidly broken down to the active metabolite psilocin [9, 10, 12] which likely exerts its psychedelic effects through agonism at 5-HT_2A_ G-protein coupled receptors [13, 14]. This receptor activity causes the downstream release of neurotransmitters that contribute to the phenomenology of the hallucinogenic experience, among other effects (promotion of neurogenesis, decreased inflammation) [15]. One or a few administrations of high-dose psilocybin, combined with psychotherapy, i.e., “psilocybin therapy”, demonstrates preliminary clinical efficacy in a growing number of mental health conditions including anxiety, depression, obsessive compulsive disorder, and substance use disorders [16–20]. Preliminary efficacy of this approach must be understood in the context of clinical trials with small sample sizes, homogenous participant populations, and the challenge of employing a robust control condition.

Clinical trials using psilocybin therapy to treat AN are currently underway (NCT05481736, NCT04505189, NCT04052568). As people with AN have unique medical vulnerability, the safety and tolerability of this proposed treatment in this population is of utmost importance. Malnutrition, or the restriction of energy intake relative to requirements leading to a significantly low body weight in the context of age, sex, developmental trajectory, and physical health, is a hallmark of AN [21]. Before treatment and weight restoration, patients with AN have lower fat mass, fat-free mass, and bone mineral density as compared with healthy controls [22]. The loss of adipose tissue in malnutrition represents the loss of a high-density energy storage mechanism that is also responsible for the secretion of hormones to regulate appetite, insulin sensitivity, and thermogenesis, among others [22, 23]. Malnutrition has variable effects on the bioavailability, metabolism, efficacy and clearance of drugs, though malnutrition has such wide-reaching physiologic implications that predicting altered pharmacokinetic and pharmacodynamic properties of psilocybin in this setting remains a challenge [24].

While no study has explicitly examined the pharmacokinetic and pharmacodynamic properties of psilocybin in the setting of malnutrition, studies point to efficacy and safety regardless of body weight. In post hoc analyses of studies with both body weight-adjusted and fixed dosing of psilocybin administrations across a wide range of body weight (49 kg to 113 kg), body weight did not significantly impact subjective drug effects or risk for adverse events [25]. Another open-label study administering escalated oral dosages of 0.3, 0.45, and 0.6 mg/kg in healthy adults did not find serious adverse effects, even in the 0.6 mg/kg dose which is larger than in standard therapeutic dosing protocols [9]. In a randomized, double-blind crossover trial of psilocybin in 51 cancer patients with life-threatening diagnoses, both low dose (1 or 3 mg/70 kg) and high dose (22 or 30 mg/70 kg) administrations were safe, with expected, transient increases in heart rate and blood pressure. While this clinical trial did not report BMI or nutritional status of participants, cancer is a well-known cause of malnutrition and cachexia and likely mimics aspects of the altered physiology often present in individuals with AN. Finally, the only published safety and feasibility trial of psilocybin administration in participants with AN (*N* = 10, average body mass index [BMI] 19.7 kg/m2) reported no serious adverse effects following administration of 25 mg of psilocybin [26]. While these studies point toward the safety of high-dose psilocybin in those with malnutrition secondary to AN, conservative dosing strategies and/or dose escalation protocols could be considered in more severely ill participants.

The purpose of this review is to describe how the common physiologic adverse effects of psilocybin (elevated heart rate and blood pressure, QT prolongation, nausea, headache, and lightheadedness) may impact medical complications experienced by individuals with AN in clinical trials of psilocybin therapy. The existing evidence for the use of psilocybin therapy in those with AN will be explored, including reported adverse effects. Subsequently, we provide recommendations for risk mitigation strategies unique to this population. In all, people with AN are likely to tolerate psilocybin therapy comparably to other clinical populations. Minor, thoughtful modifications to existing safety protocols are likely to address the unique physiologic vulnerabilities seen in people with AN.

## Potential physiologic adverse effects and risk mitigation strategies

### Cardiac complications

#### Hypertension and Tachycardia

##### Psilocybin risks and mechanisms

Hypertension and tachycardia are known effects of psilocybin administration thought to be driven by sympathetic activation [27]. Through metabolic release of its benzene ring, psilocybin may be transformed into dopamine and subsequently norepinephrine and epinephrine, although the exact mechanism of sympathetic activation is not fully understood [28, 29]. Most clinical trials of psilocybin therapy cite transient increases in blood pressure and heart rate in participants during drug intoxication; thus, those with hypertension at baseline are typically excluded from participation. In the largest clinical trial of psilocybin therapy to date in *N* = 233 individuals with treatment-resistant depression (Goodwin et al.), 19% of participants who received 25 mg psilocybin experienced isolated, out-of-range, clinically important vital sign changes (*n* = 15), compared to 32% who received 10 mg psilocybin (*n* = 24). The vital sign abnormalities were not characterized further [30]. In the second largest clinical trial of psilocybin therapy to date in *N* = 104 individuals with major depressive disorder, Raison et al., reported no clinically significant changes in vital signs [31]. In contrast, a smaller clinical trial of psilocybin therapy in *N* = 24 participants with major depressive disorder revealed an increase in heart rate above 110 beats per minute in three participants, and an elevated diastolic blood pressure above 100 millimeters of mercury (mmHg) in one participant [32].

##### Risks and mechanisms in AN

The increases in blood pressure and heart rate noted with psilocybin administration are unlikely to worsen existing vital sign abnormalities in individuals with AN who predominantly present with bradycardia and hypotension secondary to malnutrition. Individuals with long-standing AN or more severe malnutrition may have baseline heightened sympathetic tone in addition to reduced left ventricular mass and myocardial atrophy, placing them at theoretical risk of transient reductions in left ventricular function and decreased cardiac output [33, 34]. In clinical trials of psilocybin therapy in medically vulnerable populations, significant cardiac compromise has not been reported [17, 26, 35]. For example, in a study of participants with life-threatening cancer, of those who received 25 mg of psilocybin (*n* = 14), 76% showed transient increases in heart rate and blood pressure (average maximum heart rate 71 beats per minute at approximately 300 min post-dose; average maximum blood pressure 142/83 mmHg around 180 min post-dose. All deemed to be clinically non-significant) [17]. The only published safety and feasibility trial of psilocybin administration in participants with AN (*N* = 10)reported no clinically significant changes in heart rate or blood pressure during administration of 25 mg of psilocybin [26]. While both psilocybin and AN have effects on sympathetic activation and therefore heart rate and blood pressure, the opposing directionality of these effects will likely not lead to significant adverse effects in individuals with AN.

#### QT interval prolongation

##### Psilocybin risks and mechanisms

Characterizing a novel drug’s impact on QT and rate corrected QT interval (QTc) is required by the U.S. Food and Drug Administration to assess the risk of pro-arrhythmic effects. A study examining psilocybin’s effect on corrected QT intervals (QTc) in 12 healthy adult participants using a dose escalation strategy up to 0.6 mg/kg (dose range 19–59 mg) did not show significant changes in QTc intervals [36]. At a clinical dose of 25 mg, the mean QTc change was 2.1 (SD 6.6) milliseconds [36]. In Goodwin et al., two participants in the 25-mg psilocybin dose group (*n* = 79) had a significant change in QTc from baseline of > 60 milliseconds [31].

##### Risks and mechanisms in AN

Prolonged QTc intervals with cardiac consequences, including progression to ventricular arrythmia and sudden death, is a cause of mortality in individuals with AN [37]. Prolonged QTc intervals in patients with AN appear secondary to modifiable risk factors including electrolyte abnormalities such as hypokalemia or medication-induced prolongation rather than inherent vulnerability in individuals with AN [38–40]. In fact, one meta-analysis did not find QTc differences in individuals with AN relative to controls [41]. Taken together, the risk of QTc interval prolongation with psilocybin administration in patients with AN is likely low if other risk factors are minimized. Peck et al. did not find significant electrocardiogram (ECG) changes in individuals with AN following a single dose of 25 mg of psilocybin [26].

##### Recommendations

See Table 1 for overview. While cardiac adverse events are likely to be rare, given their potential severity we recommend several strategies to mitigate potential risks. Collecting vital signs, including heart rate, blood pressure, and orthostatic changes, at medical screening and prior to psilocybin dosing allows research staff to intervene on those meeting widely accepted medical hospitalization criteria [42, 43]. Monitoring vital signs at regular intervals during dosing ensures that clinically significant changes are documented and treated, if necessary. Medications which can mitigate dangerous and/or persistent tachycardia or hypertension following psilocybin administration are typically available in case of emergency, such as clonidine and nitroglycerin, among others [31]. Caution should be exercised if administering these medications to participants with known baseline hypotension and/or bradycardia. Drawing serum electrolytes at study entry and 24–48 h prior to each psilocybin administration allows for correction of electrolyte abnormalities, thereby reducing the risk of prolonged QTc and life-threatening arrhythmias. Baseline ECG, as above, can identify patients with prolonged QTc intervals or arrhythmias of clinical significance. ECG monitoring is a safe and cost-effective monitoring tool to optimize safety and minimize risks to participants both prior to and during psilocybin administration. Most clinical trials of psilocybin therapy exclude patients with significant ECG anomalies or those with a significant cardiac history at the investigator’s discretion. As clinical trials gravitate toward the inclusion of more severely ill populations, we expect further refinement of these inclusion and exclusion criteria.

### Headaches

#### Psilocybin risks and mechanisms

Headaches are one of the most common reported adverse effects experienced following psilocybin administration. Goodwin et al. reported that 24% of participants who received 25 mg of psilocybin reported headache, as did 15% of participants who received 10 mg of psilocybin [30]. 16% of participants who received 1 mg of psilocybin (a sub-perceptual dose) also reported headache [30]. In Raison et al., 66% of participants who received 25 mg of psilocybin reported headache versus 24% in the group who received niacin [31]. The precise mechanism of how psilocybin causes headaches remains unknown, although cerebral vascular effects are a possibility. Particularly in the setting of a clinical trial, dosing days may last for six to eight hours or longer with less than adequate hydration during this time, leading to dehydration and resulting headache [44]. Thus far, headaches and migraines reported in clinical trials of psilocybin therapy are reported as transient, though this has not been further characterized with a duration [26, 30].

#### Risks and mechanisms in AN

Limbic and hypothalamic dysfunction may be mechanisms underlying both the development of headache, including migraine, and the development of AN, possibly explaining the increased prevalence of headache in patients with AN [45, 46]. One study of individuals with eating disorders (sample included patients with AN and bulimia nervosa) found that among 109 participants, 84.4% experienced some type of headache, either migraine or tension headache, rates much higher than in the general population [45]. In contrast, a subsequent study in people with eating disorders and their non-eating disordered siblings did not find a significant difference in the prevalence of migraine [47]. While headaches including migraines are certainly not unique to people with AN, clinical trials of psilocybin therapy must prepare for participants with AN to experience headache as an expected adverse effect. In the first published open-label feasibility study of psilocybin therapy in females with AN, eight of ten participants reported headaches and two of ten reported migraines following a dose of 25 mg of psilocybin [26].

#### Recommendations

See Table 1 for overview. Clinical trials can consider mitigating the risk of dehydration-induced headache, particularly in a medically vulnerable population like those with AN, by requiring participants to drink electrolyte-containing beverages at regular intervals throughout psilocybin dosing sessions. Sumatriptan and other serotonergic abortive medications are contraindicated in psilocybin clinical trials given potential for drug-drug interactions at serotonergic receptors. However, acetaminophen or ibuprofen are safe pharmacologic options that may be administered either prophylactically on the morning before dosing or as needed following dosing. If not used preventatively, this pharmacologic option should be discussed with participants in the case that they do develop headache following dosing. Clinical trials might also incorporate behavioral treatments to prevent migraines and headaches for trial participants, including relaxation or biofeedback techniques, particularly for those with known history of these conditions [48]. Participants should be advised to maintain their typical daily caffeine intake for the duration of the study to avoid caffeine-withdrawal headaches. Considering the transient nature of headaches following psilocybin dosing in clinical trials thus far, the potential for headache as an adverse effect in people with AN should not be a barrier to trial enrollment.

### Nausea

#### Psilocybin risks and mechanisms

Nausea is one of the most common adverse effects reported in clinical trials of psilocybin therapy [49], with Goodwin et al. noting this adverse effect in 22% of participants and Raison et al. noting this adverse effect in 48% of participants within their respective 25 mg psilocybin groups [30, 31]. While synthetic formulations of psilocybin eliminate chitin and other compounds in raw mushrooms which impede human digestion, nausea remains a challenge in clinical trials of psilocybin therapy. Ingested orally, as is the case in most clinical trials of psilocybin therapy, psilocin also interacts with peripheral 5-HT2A receptors, which are of particularly high density in the GI tract, particularly in myenteric and submucosal neurons, enterocytes and circular muscle cells [50–52]. Activation of these receptors contributes to the contraction of smooth muscle cells, promoting both peristalsis and secretory function [53–55]. Plausibly, enhanced peripheral 5-HT2A activation may explain the psilocybin-induced nausea frequently reported in clinical trials. Similarly, action at peripheral 5-HT3 receptors, which are ligand-gated ion channels, have diverse gastrointestinal excitatory effects, including activation of myenteric reflexes, mucosal secretion, and vagal afferents that increase glutamate transmission in the brainstem [56–59]. Excessive activation of these vagal afferent 5-HT3 receptors has been well implicated in the development of nausea, and 5-HT3 receptor antagonists prevent or improve nausea symptoms [60–62]. The combined effects of psilocybin-induced activation of 5-HT2A and 5-HT23 receptors alters a complex neurochemical milieu, which could disrupt normal GI function, contributing to nausea.

#### Risks and mechanisms in AN

Nausea in individuals with AN has a multifaceted etiology and can present at different points throughout the illness. Interactions between physiologic and psychological functions can exacerbate or mitigate nausea through significant weight changes, malnutrition, eating disorder behaviors, and disrupted eating patterns. These changes often disrupt normal gastrointestinal motility and secretion of hormones, thereby contributing to delayed gastric emptying, gastroparesis, and hypersensitivity to interoceptive cues, among others, often leading to nausea in individuals with AN [63–65]. In all, nausea in AN is multifactorial but highly prevalent in various stages of the disease course.

In the phase 1, open-label feasibility study of psilocybin therapy in females with AN, three of ten participants (30%) reported mild and transient nausea as a treatment-emergent adverse effect [26]. In a qualitative exploration of 16 individuals with a history of eating disorder undergoing ceremonial ayahuasca drinking in which nausea and vomiting are expected, none cited nausea or vomiting as triggering of eating disorder behaviors or symptoms [66]. Thus far, nausea seems to be a transient and common adverse effect of classic psychedelic ingestion that is not more pronounced in individuals with AN, despite high background rates of nausea accompanying the illness.

#### Recommendations

See Table 1 for overview. For individuals with AN in a clinical trial of psilocybin therapy, several considerations can mitigate the risk of nausea. Encouraging or mandating the intake of nutrition on the morning of dosing may help to eliminate nausea precipitated by the acute effects of restrictive intake or administration of psilocybin on an empty stomach. Optimization of nutrition in advance of dosing may also help to correct some of the abnormalities that precipitate nausea, i.e., improvements in gastroparesis; however, this may not be feasible in the setting of deeply entrenched eating disorder cognitions and behaviors. Other strategies may include optimization of a constipation regimen prior to dosing. Any medications utilized to mitigate symptoms must be carefully checked with study exclusionary medications to minimize risk of drug-to-drug interactions including synergistic QT prolongation, for which patients with AN are at elevated risk [34]. Treatment with antiemetics such as ondansetron, prochlorperazine, and diphenhydramine are typically contraindicated in clinical trials of psilocybin therapy due to potential for drug interactions or blunting of the serotonergic effects of psilocybin [67]. Integrative therapies should be explored in the context of treatment-emergent nausea, including evidence-based interventions like aromatherapy and mind-body therapies [68]. These techniques should not disrupt the primary intervention. In all, the risk of nausea in the context of clinical trials of psilocybin therapy for individuals with AN should not present a barrier to enrollment or completion of study activities.

### Lightheadedness

#### Psilocybin risks and mechanisms

Lightheadedness or dizziness is an effect of psilocybin consumption although the etiology of this adverse effect is not well understood. Among 79 participants who received 25 mg psilocybin for treatment-resistant depression in the study by Goodwin et al., 6% reported dizziness during or following administration [30]. Dizziness (which we will use interchangeably with lightheadedness) plausibly results from the pharmacological effects of psilocybin, which globally acts as an excitatory sympathomimetic compound (i.e., raising blood pressure and heart rate, see those sections for additional details) [69]. Activation of the peripheral serotonin receptors specifically drives excitation of vagal afferent fibers which can contribute to the vasovagal reflex. Similarly, in those with sensitive vasovagal reflexes or prone to orthostasis, the sudden increase in vagal tone and increased sympathetic drive from psilocybin consumption could yield lower ventricular filling. In turn, cardiac contraction around a cardiac chamber with reduced venous return could drive an acute increase in vagal tone causing lightheadedness and vasovagal syncope [70]. Furthermore, traditional psilocybin administration in clinical trials are accompanied by prolonged periods without caloric intake due to both an altered state of consciousness and the common side effect of nausea. While data is lacking, hypoglycemia during these fasting periods could theoretically contribute to lightheadedness and/or the predisposition to syncope [26].

#### Risks and mechanisms in AN

Dizziness is common in patients with AN with a multifactorial etiology. Orthostasis and hypoglycemia, among other etiologies, likely account for most episodes of dizziness among patients with AN.

Dizziness from orthostasis: Orthostasis is a common vital sign instability noted in those with malnutrition secondary to AN, which can manifest as lightheadedness, dizziness, or syncope [71]. The malnourished state exacerbates autonomic dysregulation through increased vagal tone, impairing the cardiovascular system’s ability to adequately regulate blood pressure and vascular tone, particularly in response to positional changes [72].

Dizziness from hypoglycemia: The caloric deprivation and low carbohydrate stores in individuals with AN may lead to glucometabolic dysregulation and hypoglycemia. A shift to gluconeogenesis is often required to maintain glucose homeostasis [73, 74]. The brain, a highly glucose-dependent organ, is particularly sensitive to episodes of hypoglycemia, resulting in anxiety, dizziness, disorientation, and in end stages, seizures and sudden death [75].

Given these susceptibilities, the introduction of psilocybin to this metabolic and nervous system milieu may amplify the risk of dizziness. In the phase 1, open-label, feasibility study of psilocybin therapy for AN by Peck et al., two of ten participants (20%) reported dizziness as a treatment-emergent adverse effect and two participants experienced hypoglycemia on dosing day that resolved within 24-hours [26]. Participants in this trial were required to eat breakfast prior to psilocybin administration, both in anticipation of a period of prolonged fasting and low glucose stores as part of the illness.

**Recommendations**: See Table 1 for overview. For individuals with AN in a clinical trial of psilocybin therapy, several considerations can mitigate the risk of dizziness resulting from either orthostasis or hypoglycemia. Encouraging and even mandating the intake of nutrition on the morning of dosing may help reduce the risk of hypoglycemia and subsequent dizziness or syncope. The nutritional content of this pre-dosing nutrition may be a consideration: meals with higher fat content, as opposed to carbohydrates alone, may prevent the post-prandial insulin surge which can precipitously drop serum glucose levels [74, 76]. Indeed, nutritional intake at regular intervals during psilocybin therapy may be warranted to prevent periods of hypoglycemia. While mindful of not disrupting the therapeutic process and altered state of consciousness, beverages with electrolytes and calories or small snacks may mitigate the risks of hypoglycemia during psilocybin therapy. The benefits of this strategy are two-fold: adequate hydration also mitigates orthostasis, for which patients with AN are at higher risk. By capturing orthostatic symptoms and vital signs at baseline, the study team will know which trial participants may be at risk for orthostasis. During psilocybin administration and acute effects, when the participant undergoes positional changes (laying down to standing, ambulating to the restroom during psilocybin therapy, etc.), research staff should provide ambulatory support and encourage slow positional changes. In all, the risk of lightheadedness during clinical trials of psilocybin therapy for individuals with AN should not present a barrier to enrollment or completion of study activities with careful risk mitigation strategies.

**Table 1:** Risk mitigation strategies

Adverse Effect	Risk Mitigation Strategy
Cardiac: • Hypertension • Tachycardia • Prolonged QT interval	1) Obtain vital signs: heart rate, blood pressure, and orthostatic vital signs a) When: at initial medical screening, 24–48 h prior to psilocybin dosing, just prior to psilocybin dosing, and every 1–2 h after administration b) Participants with medical instability requiring medical intervention should not be dosed c) Participants who develop dangerous and/or persistent hypertension and/or tachycardia during psilocybin administration may require rescue medications for immediate stabilization (consider clonidine, nitroglycerin, propranolol) 2) Measure serum electrolytes a) When: at study entry and 24–48 h prior to each psilocybin administration 3) Obtain ECG a) When: at medical screening b) Participants with abnormal ECGs should be evaluated for participation by a cardiologist or other appropriate medical provider
Headache	1) Require maintenance of typical daily caffeine intake during study 2) Require intake of nutrition and electrolyte-containing beverages* a) When: prior to psilocybin dosing and at regular intervals throughout psilocybin dosing day 3) Utilization of acetaminophen or ibuprofen a) When: prophylactic on the morning before dosing or as needed following dosing 4) Consider incorporation of relaxation or biofeedback techniques 5) For participants with migraines, triptans are contraindicated around psilocybin administration given risk of serotonin syndrome
Nausea	1) Require intake of nutrition* a) When: prior to psilocybin dosing and at regular intervals throughout psilocybin dosing day 2) Optimize constipation/bowel regimen through medication and diet a) When: weeks prior to dosing 3) Behavioral strategies to mitigate nausea a) When: potentially during dosing if nausea occurs 4) Ondansetron, prochlorperazine, and diphenhydramine are contraindicated
Dizziness	1) Require intake of nutrition and electrolyte-containing beverages* a) When: prior to psilocybin dosing and at regular intervals throughout psilocybin dosing day 2) Obtain orthostatic vital signs a) When: at screening visit and 24 to 48 h prior to doing day b) Patients with symptomatic orthostasis should not be dosed. 3) Staff to provide ambulatory support during any participant positional changes a) When: during dosing day

*Recommended approach: Calculate approximate individual fluid requirements for the duration of the dosing day (4 ml/kg/hr for the participant’s first 10 kg body mass, 2 ml/kg/hr for participant’s second 10 kg body mass, and 1 ml/kg/hr for each kilogram of body mass remaining). Encourage at least 25% of this intake prior to psilocybin administration with the remaining intake completed throughout the dosing day and prior to participant departure. Fluids should be calorie and electrolyte containing (e.g. sports drink, milk, etc.). Optimally protein and fat should be included, either through a beverage (e.g. milk) or additional snack (bar, trail mix, etc.)

## Conclusion

Common physiologic adverse effects from psilocybin have parallels with symptomatology often present in individuals with AN. Existing evidence for the use of psilocybin therapy in patients with AN, although limited (total *N* = 10), revealed rates and severity of adverse effects similar to those experienced by other clinical populations receiving psilocybin therapy. Despite their unique medical vulnerabilities, patients with AN are likely to tolerate psilocybin therapy with careful medical screening, medical management, and tailored risk mitigation strategies. Anticipatory guidance about the more common physiologic adverse effects from psilocybin administration is warranted. Larger clinical trials with more participant diversity will help deepen our understanding of safety and tolerability of psilocybin therapy in this population and to optimize this intervention in preparation for real-world clinical settings.

## Data Availability

No datasets were generated or analysed during the current study.

## Abbreviations

AN: Anorexia nervosa
BMI: Body mass index
DMN: Default mode network
QTc: Corrected QT interval
ECG: Electrocardiogram
